# First evidence of a hyperdirect prefrontal pathway in the primate: precise organization for new insights on subthalamic nucleus functions

**DOI:** 10.3389/fncom.2013.00135

**Published:** 2013-10-10

**Authors:** Simon Nougaret, Julie Meffre, Yann Duclos, Emmanuel Breysse, Yann Pelloux

**Affiliations:** Institut de Neurosciences de la Timone, UMR7289 CNRS, Aix-Marseille Université, Marseille, France

**Keywords:** subthalamic nucleus, prefrontal cortex, hyperdirect pathway, labeling, deep brain stimulation, anatomy, limbic processes

The direct connections from the cortex to the subthalamic nucleus (STN), the so-called hyperdirect pathway, is known for the cortical motor areas and plays a top–down executive control on basal ganglia (BG). However, little was known regarding the projections onto the STN from anterior and ventral prefrontal regions involved in more integrated functions such as decision making or reward related processes.

The large-scale study by Haynes and Haber aimed to trace the hyperdirect pathway from different territories of the prefrontal cortex and motor areas to determine the levels of convergence and segregation of these projections onto the different subterritories of the STN. Their first objective was to delineate all frontal inputs to the STN in monkeys, extending to primate those already described in rodents (Berendse and Groenewegen, 1991). They impressively targeted many areas constitutive of four prefrontal regions: ventromedial prefrontal cortex (vmPFC), orbitofrontal cortex (OFC), dorsal anterior cingulate cortex (dACC) and dorsal prefrontal cortex (dPFC), and established that all of them project onto the STN. These cortices are differentially involved in cognitive, motivational and emotional processes. Do these distinct information funnel in the STN or remain processed separately by STN subterritories? The second objective was to delineate a limbic STN based on the topography of the projections from areas of the vmPFC, OFC, and dACC, involved in reward-related processes. The authors mapped the limbic part of the STN from the medial tip of the nucleus to the lateral part of the LH. However, since not all areas from the vmPFC and OFC and other limbic cortices have been investigated, the study does not allow to assess the exact extent of this limbic STN. Finally, the authors examined the convergence and/or segregation of cortico-STN fibers from motor, cognitive and limbic cortical areas. The central part of the STN receives overlapping projections from the majority of labeled cortical afferences. However, motor projections to the dorsal lateral extremities seem to be isolated from those forming the limbic territories, located at the medial tip of the nucleus.

## What is the functional relevance of the hyperdirect pathway?

The BG are known to be involved in motor, associative and limbic functions. The convergence of these different types of information onto this set of subcortical structures supports its involvement in action selection. In the circuitry of the BG, the signal conveyed through the cortex-STN hyperdirect pathway reaches the BG outputs before information conveyed through the other pathways. One hypothesis suggests that the STN is able to integrate information and to act as a decisional threshold to permit or not to perform the action (Frank et al., 2007). The STN would send a global NoGo signal to exert a consequent inhibition on thalamo-cortical activity, providing a high cognitive control in the face of conflict. Alternatively, Peron et al. (2013) have proposed that the STN organizes emotional response patterns among cortical and subcortical limbic structures. Haynes and Haber (2013) provide the first evidence, to our knowledge, for a “limbic” hyperdirect pathway between the OFC/vmPFC and the STN in primates. Functional connections between the STN and the medial prefrontal cortex (mPFC) have been previously characterized in rats in a cognitive task, showing that mPFC/STN disconnection impairs attentional processes and elicits perseverative responding (Chudasama et al., 2003). Alternatively, the functional connectivity of the OFC and the STN remains to be characterized. Separately, OFC and STN inactivations result in similar motivational impairments and hold similar electrophysiological properties such as encoding the relative value of the reward (Tremblay and Schultz, 1999; Lardeux et al., 2013). In addition, these two structures are involved in inhibition control as evidenced by the inability of OFC or STN lesioned rats to repress their ongoing behavior in a stop task (Eagle and Baunez, 2010; Baunez and Lardeux, 2011). To date, functional commonalities could have been attributed to transmission from the OFC to the STN through the classical indirect pathway (*via* the striatum and the external part of the globus pallidus or the ventral pallidum). Now, the monosynaptic connection between the OFC and the STN, revealed in this study, might be important to explain the effects of the STN manipulations.

Integration of the diverse cortical inputs within the STN is supported by the morphology of its neurons. Indeed, their dendritic fields, particularly at the center of the nucleus, can spread broadly in the structure (Yelnik and Percheron, 1979). At each pole, only the hemifield toward the other pole remains, offering ample surface for the convergence of multiple inputs while preserving the continuous gradient of cortical projections. Topography at the extremities is likely to be conserved if the proximal inputs, originating predominantly from one frontal area, remain more efficient than those more distal, as expected from the decremental propagation of the signal along the dendritic tree. Subregional specificity is not supported by electrophysiological data in monkeys, showing that STN neurons respond to stimuli predictive of reward or reward itself independently of their location within the nucleus (Darbaky et al., 2005) but this remains to be investigated for other functions subserved by the prefrontal cortex.

Regional specialization of the STN has led clinicians to target preferentially the dorsolateral part of this structure, being considered the motor territory, for therapeutical deep brain stimulation (DBS) application for Parkinson's disease. In contrast, DBS of the ventromedial STN has been successfully applied in the treatment of obsessive-compulsive disorder (OCD) (Mallet et al., 2008). Stimulation at this level decreases the functional activity of the prefrontal cortex (Le Jeune et al., 2010). Because OCD is associated with increased OFC functional activity, the therapeutical benefits of STN DBS could result from an antidromic effect on cortical metabolism. Nevertheless, the same consequences on prefrontal activity could be detrimental in some PD patients, causing behavioral complications.

In conclusion, Haynes and Haber (2013) provide a novel insight of the mapping of prefrontal connections to the STN. Improved imaging and implantation techniques would greatly help clinicians to reproducibly and precisely implant DBS electrodes in STN subregions. This improvement will enhance the therapeutical efficacy of STN DBS and possibly reduce its side effects.
